# A case of malignant hypertension as a presentation of atypical hemolytic uremic syndrome 

**DOI:** 10.5414/CNCS110901

**Published:** 2023-04-28

**Authors:** Chiaki Omiya, Kenichi Koga, Keisuke Nishioka, Akira Sugawara, Yuka Sugawara, Yoko Yoshida, Yoichiro Ikeda, Kensei Yahata

**Affiliations:** 1 Department of Nephrology, Osaka Red Cross Hospital, Osaka, and; 2 Department of Nephrology and Endocrinology, The University of Tokyo Hospital, Tokyo, Japan

**Keywords:** atypical hemolytic uremic syndrome, complement-related proteins, malignant hypertension, thrombotic microangiopathy

## Abstract

Introduction: Malignant hypertension (mHTN) damages multiple target organs, including the kidneys. mHTN has been regarded as one of the causes of secondary thrombotic microangiopathy (TMA); however, a high prevalence of complement gene abnormalities was recently reported in cohorts of mHTN. Case report: We herein describe a 47-year-old male who presented with severe hypertension, renal failure (serum creatinine (sCr): 11.6 mg/dL), heart failure, retinal hemorrhage, hemolytic anemia, and thrombocytopenia. Renal biopsy findings were consistent with acute hypertensive nephrosclerosis. The patient was diagnosed with secondary TMA associated with mHTN. However, his previous medical history of TMA of unknown origin and family history of atypical hemolytic uremic syndrome (aHUS) suggested as aHUS presenting mHTN, and genetic testing revealed a pathogenic C3 mutation (p.I1157T). The patient required plasma exchange and hemodialysis for 2 weeks and was able to withdraw from dialysis by antihypertensive therapy without eculizumab. Renal function gradually improved to a sCr level of 2.7 mg/dL under antihypertensive therapy for 2 years after the event. There was no recurrence, and renal function was preserved throughout a 3-year follow-up. Discussion: mHTN is a common presentation of aHUS. In cases of mHTN, abnormalities in complement-related genes may be involved in the development of the disease.

## Introduction 

Malignant hypertension (mHTN) is a critical condition that induces acute target organ damage to the kidneys, brain, heart, and large blood vessels. Microvascular damage and pressure natriuresis caused by severe hypertension result in ischemia of the renovascular bed and the paradoxical activation of the renin-angiotensin-aldosterone system (RAAS). The stimulation of RAAS promotes further increases in blood pressure through high levels of circulating angiotensin II, leading to more vascular damage [1]. Thrombotic microangiopathy (TMA) occasionally develops in mHTN via endothelial damage caused by shear stress and mHTN has been regarded as a cause of secondary TMA [2]. A high prevalence of complement-related gene abnormalities was recently reported in cohorts of TMA associated with mHTN [3, 4]. Although the role of the complement system in mHTN remains unclear, there is increasing evidence to support a relationship between mHTN and complement dysregulation [5]. We herein report a case of aHUS with a pathogenic mutation in C3 (p.I1157T) that presented as mHTN. 

## Case report 

A 47-year-old Japanese man presented to the emergency department with dyspnea. He had a markedly elevated blood pressure (205/129 mmHg) and severe renal failure (serum creatinine (sCr): 11.6 mg/dL), and was referred to our department for further management. The patient had a history of hematuria and acute renal failure at 8 years of age, and was diagnosed with TMA of unknown origin. He recovered without specific treatment and his renal function had not been followed up. The patient started having annual medical check-ups several years ago, and hypertension (blood pressure of ~ 180/100 mmHg) and mild renal insufficiency (sCr 1.07 mg/dL 1 year before admission) were detected; however, he did not visit a doctor. The patient had a history of drinking 1,500 mL of beer and smoking 20 cigarettes per day for 27 years. There was a family history of atypical hemolytic uremic syndrome (aHUS) with a *C3* gene mutation (p.I1157T) in his niece, who had several episodes of aHUS in her childhood. Although aHUS recurred when she was 28 years old, she promptly recovered after eculizumab treatment 

The patient was awake and alert on admission. His height was 177 cm, and body weight was 84.7 kg. Blood pressure was 205/129 mmHg, pulse rate 92 beats/min, and body temperature 36.8 °C. He had oliguria, and a physical examination revealed coarse crackles in both lung fields and mild leg edema. Chest X-ray showed decreased permeability in both lower lung fields, and diffuse myocardial hypertrophy was noted on cardiac echocardiography. The plasma level of brain natriuretic peptide increased to 1,282.7 pg/mL (normal range 0 – 18.4 U/L); however, the left ventricular ejection fraction was preserved (65%). These findings indicated acute hypertensive decompensated heart failure. A neurological examination revealed no focal signs, and there were no findings of cerebral infarction or microhemorrhage on head magnetic resonance imaging. Fundoscopy demonstrated stage III on the Keith-Wagener classification in both eyes, including findings of retinal hemorrhage and soft exudates, which were compatible with mHTN. A laboratory examination revealed the following (Table 1): sCr, 11.57 mg/dL (normal range 0.65 – 1.07 mg/dL); blood urea nitrogen, 106 mg/dL (normal range 8 – 20 mg/dL); serum uric acid, 9.8 mg/dL (normal range 3.7 – 7.8 mg/dL); lactate dehydrogenase, 816 U/L (normal range 124 – 222 U/L); hemoglobin, 10.3 g/dL (normal range 13.7 – 16.8 g/dL); platelet count, 52,000/μL (normal range 158,000 – 348,000/μL). The minimum levels of hemoglobin and the platelet count during the clinical course were 9.0 g/dL and 51,000/μL, respectively. Blood transfusion was not performed before or after admission. A peripheral blood smear revealed 1% schistocytes (normal range < 1%). Urinalysis showed proteinuria (6.23 g/gCr), 3+ hematuria (10 – 19 erythrocytes/high-power field), and various sediments. C3 and C4 levels were 82 mg/dL (normal range 73 – 138 mg/dL) and 31 mg/dL (normal range 11 – 31 mg/dL), respectively. The level of haptoglobin was below detectable ranges. A disintegrin-like and metalloprotease with thrombospondin type 1 motifs 13 (ADAMTS-13) activity was 52% (normal range > 78%), and an ADAMTS-13 inhibitor was not detected. Plasma renin activity and the plasma concentration of aldosterone were 13 ng/mL/h (normal range 0.3 – 2.9 ng/mL/h) and 285 pg/mL (normal range 29.9 – 159 pg/mL), respectively. 

**Figure 1:**
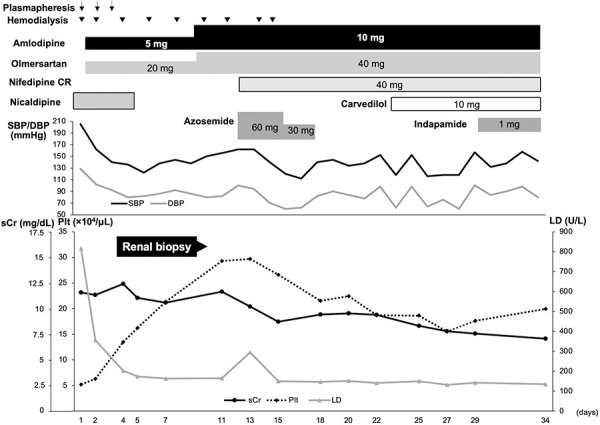
Clinical course. SBP = systolic blood pressure; DBP = diastolic blood pressure; sCr = serum creatinine; Plt = platelet count; LD = lactate dehydrogenase.

Based on these findings, the patient was diagnosed with secondary TMA with mHTN. The continuous intravenous infusion of nicardipine was started as initial therapy for severe hypertension (Figure 1). His previous medical history of TMA and family history of aHUS suggested that mHTN developed as a part of aHUS. Plasma exchange was performed for 3 days until improvements were detected in hematological abnormalities. However, eculizumab was not administered because the patient expressed concerns about adverse effects. Hemodialysis was required for oliguria and uremia, but was withdrawn within 2 weeks. Severe hypertension was treated with multiple antihypertensive drugs, including a Ca blocker (CCB) and angiotensin receptor blocker (ARB). 

**Figure 2:**
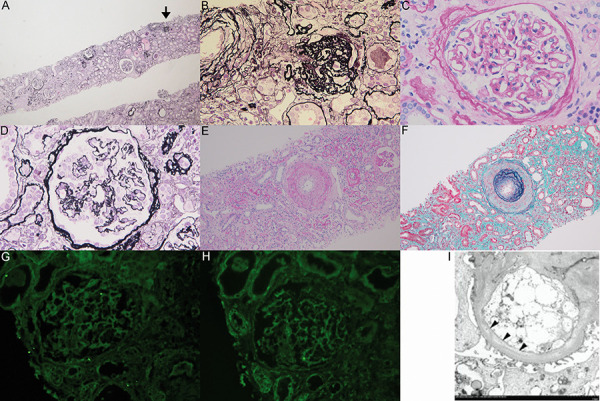
Renal biopsy findings. A: Ischemic glomeruli and global sclerotic glomeruli (arrow) were observed, and tubular atrophy and interstitial fibrosis were detected in 30% of the area (periodic acid methenamine staining, original magnification × 40). B: A globally sclerotic glomerulus shown in (A) indicated by an arrow (periodic acid methenamine staining, original magnification × 400). C, D: In glomeruli, there were mild ischemic changes, but no thrombi (C: periodic acid-Schiff staining, original magnification × 400, D: periodic acid methenamine staining, original magnification × 400). E, F: An interlobular artery exhibited “onion skin” with a narrowing lumen (E: periodic acid-Schiff staining, original magnification × 100, F: Elastica-Masson staining, original magnification × 100). G, H: There were no deposits of IgG (G) or C3 (H) on direct immunofluorescence microscopy (original magnification × 200). I: Slight subendothelial edema (arrowheads) was observed on electron microscopy (original magnification × 18,000).

Renal biopsy was performed on the 10^th^ day after onset. Among the 17 glomeruli examined, 3 were globally sclerosed. Although there were no apparent thrombi in glomeruli, ischemic changes were observed (Figure 2A – D), which were presumably associated with arteriolar narrowing due to intimal thickening (Figure 2E, F). Tubular atrophy and interstitial fibrosis were observed in 30% of the area (Figure 2A). Direct immunofluorescence microscopy showed no deposits of immunoglobulins or complements (C3 and C4) (Figure 2G, H). Slight subendothelial edema was detected on electron microscopy (Figure 2I). These pathological findings were consistent with acute hypertensive nephrosclerosis and also aHUS. After the withdrawal of dialysis, the level of sCr slowly decreased (7.5 mg/dL), and the patient was discharged on the 29^th^ day. A genetic analysis was subsequently conducted at the aHUS office of Tokyo University, and the same pathogenic mutation in *C3* (c.3470T>C, p.I1157T) as that of his niece was identified. No other pathogenic mutations in complement-related genes or the anti-CFH antibody were detected. Renal function gradually improved to a sCr level of 2.7 mg/dL under anti-hypertensive therapy for 2 years after the event. There was no recurrence and renal function was preserved throughout the 3-year follow-up. 

## Discussion 

mHTN induces acute target organ damage to the brain, heart, kidneys, and large blood vessels due to high blood pressure. The kidney is a major target organ in mHTN, and this renal complication is described as acute hypertensive nephrosclerosis. mHTN causes TMA due to endothelial damage induced by shear stress. Our patient was initially diagnosed with secondary TMA complicated by mHTN. However, genetic testing revealed a pathogenic mutation in C3 (p.I1157T), suggesting aHUS presenting as mHTN. 

A high prevalence of genetic defects in complement regulatory genes was recently reported in a cohort of severe and malignant hypertension [3, 4]. Timmermans et al. [3] investigated the frequency of complement gene abnormalities in a cohort of hypertension-associated TMA, and 8 out of 17 patients had known complement gene abnormalities. In addition, they discussed the possible roles of complement gene abnormalities in the development of mHTN based on an ex vivo study, in which vascular endothelial cells were incubated with the serum from patients with hypertension-associated TMA, and C5b9 was significantly induced on endothelial cells. Based on these findings, a certain proportion of hypertension-induced TMA may be classified as complement-mediated aHUS [6]. 

TMA caused by mHTN has long been categorized as secondary TMA because hypertension per se may cause acute significant endothelial damage [6]. However, recent studies suggested that hypertension acts as one of the triggers for complement activation in aHUS patients [2, 7]. The present case had no apparent triggers for the recurrence of aHUS, such as infections, vaccinations, drugs, and surgical treatments that activate the complement system, other than uncontrollable severe hypertension, which may activate the complement system and cause a vicious cycle of hypertension, leading to clinically significant endothelial damage. Although it is important to establish whether patients with TMA and severe hypertension have mutations in complement-related genes before the initiation of treatments, the genetic testing for aHUS is not generally performed because of the lack of awareness that aHUS sometimes presents as mHTN [3]. 

Renal biopsy may be helpful for distinguishing acute hypertensive nephrosclerosis from other forms of TMA or other renal diseases. The typical lesion of acute hypertensive nephrosclerosis is intimal edema in the small arteries, and fibrinoid necrosis may also be detected, particularly in severe cases [8]. Another characteristic lesion is an onion skin lesion, indicating fibroelastic intimal thickening with reduplication of the internal elastic lamina. Glomerular lesions are generally not as significant as those of small arteries [8]. TMA other than acute hypertensive nephrosclerosis often exhibits more prominent glomerular lesions, such as the occlusion of capillary lumina with subendothelial edema and thrombi, or capillary congestion (glomerular paralysis) [9]. Renal biopsy may distinguish acute hypertensive nephrosclerosis from other forms of TMA; however, difficulties are associated with differentiating between acute hypertensive nephrosclerosis with and without complement gene mutations based solely on renal biopsy findings. Cavero et al. [2] examined the renal biopsy findings of aHUS complicated with mHTN, and showed that the typical lesions of aHUS complicated with mHTN were onion skin lesions and interstitial fibroses, which were identical to those of mHTN without complement gene abnormalities. However, they did not report the specific lesions observed only in mHTN with complement abnormalities leading to the diagnosis of this condition. 

Timmermans et al. [3] proposed evaluating the ex vivo formation of C5b9 on microvascular endothelial cells to identify complement defects in patients presenting with TMA and severe hypertension. However, this assay is currently not available in clinical settings. 

Epidemiological considerations may be useful for understanding the significance of complement gene abnormalities in mHTN. The aforementioned Dutch cohort of mHTN also included abnormalities in the *C3* gene [3], suggesting that *C3* gene abnormalities are a risk factor for the development of mHTN. In the Japanese cohort including 45 patients with aHUS, C3 mutations were identified in 36% of patients [10], and the renal prognosis of the C3 mutation group was favorable, particularly in patients with the *C3* p.I1157T variant [11]. We don’t have solid evidence to show this *C3* mutation causes mHTN at the onset of aHUS. To evaluate the significance of the *C3* p.I1157T variant in mHTN, the prevalence of this mutation in large cohorts of mHTN needs to be investigated in Japan. A fundoscopic examination has not always been performed on patients with aHUS, which may have led to the risk of mHTN being underestimated in aHUS with this variant. 

The diagnosis of mHTN caused by aHUS is critical because both mHTN and aHUS cause TMA, and the only difference between them is the presence of severe hypertension leading to acute and overt endothelial damages which are usually diagnosed by the presence of fundoscopic abnormalities. Hypertension is usually accompanied by renal failure and sometimes becomes severe; however, the fundoscopic examination is not always done in the clinical setting of treating aHUS. The limitation to evaluate the presence of mHTN in aHUS patients might be the accurate diagnosis of fundoscopic abnormalities. 

Since eculizumab may markedly improve the prognosis of patients with aHUS [12], it is important to differentiate TMA associated with complement gene abnormalities from that without. As discussed above, this differentiation is often difficult in TMA with mHTN; however, the recurrence of TMA or a family history of aHUS suggests the involvement of complement-related genes. In these cases, treatments for aHUS, such as plasma exchange and eculizumab, may be warranted [6]. aHUS was suspected in the present case based on the family and previous medical histories, and the use of eculizumab was considered. However, the patient expressed concerns about adverse effects, and was instead treated with plasma exchange and antihypertensive drugs, including CCB and ARB. 

mHTN often occurs in untreated hypertensive patients or patients with poor compliance [13]. Although our patient was obese and hypertensive based on annual medical check-ups, he did not visit a doctor or try to improve his lifestyle. Renal biopsy findings included medial thickening in the interlobular arteries, tubular atrophy, and interstitial fibrosis, suggesting long-term pre-existing hypertension. A lack of interest in his own healthcare may have contributed to the development of mHTN. A previous study suggested a higher frequency of severe hypertension and acute hypertensive nephrosclerosis in patients with aHUS [2], indicating that educational interventions to manage risk factors may be effective for patients with abnormalities in complement-related genes. 

We found no direct evidence that abnormalities in complement-related genes played significant roles in the pathogenesis of the present case. However, we speculate that the mutation in C3 affected the pathogenesis of TMA based on increasing evidence supporting the roles of complements and their regulators in mHTN. In cases of mHTN, it is important to obtain information on previous medical and family histories of aHUS or TMA. In addition, when antihypertensive drugs fail to improve TMA in mHTN, the possibility of abnormalities in complement-related genes might be considered. 

## Funding 

There was no funding for this report. 

## Conflict of interest 

The authors have no conflict of interest to declare. 

**Table 1.** Laboratory findings on admission.

Lab test	Results (normal range)
Peripheral blood	
White blood cell count (/μL)	7,500 (3,300 – 8,600)
Red blood cell count (×10^4^/μL)	329 (435 – 555)
Schistocytes (%)	1 (< 1)
Hemoglobin (g/dL)	10.3 (13.7 – 16.8)
Platelet count (×10^4^/μL)	5.2 (15.8 – 34.8)
Blood chemistry	
Total protein (g/dL)	6.0 (6.6 – 8.1)
Albumin (g/dL)	3.8 (4.1 –5.1)
Aspartate aminotransferase (U/L)	20 (13 – 30)
Alanine aminotransferase (U/L)	10 (10 – 42)
Lactate dehydrogenase (U/L)	816 (124 –222)
Total bilirubin (mg/dL)	1.0 (0.4 – 1.5)
Direct bilirubin (mg/dL)	0.2 (0 – 0.4)
Uric acid (mg/dL)	9.8 (3.7 – 7.8)
Blood urea nitrogen (mg/dL)	106 (8 – 20)
Creatinine (mg/dL)	11.57 (0.65 – 1.07)
Sodium (mEq/L)	136 (138 – 145)
Potassium (mEq/L)	3.6 (3.6 – 4.8)
Chloride (mEq/L)	101 (101 – 108)
Ferritin (ng/mL)	787.0 (34 – 370)
Brain natriuretic peptide (pg/mL)	1,282.7 (0 – 18.4)
Plasma renin activity (ng/mL/hr)	13 (0.3 – 2.9)
Plasma aldosterone concentration (pg/mL)	285 (35.7 – 240)
Serology	
C-reactive protein (mg/dL)	0.44 (0 – 0.14)
Immunoglobulin G (mg/dL)	616 (861 – 1747)
Immunoglobulin A (mg/dL)	126 (93 – 393)
Immunoglobulin M (mg/dL)	36 (33 – 183)
Complement component 3 (mg/dL)	82 (73 – 138)
Complement component 4 (mg/dL)	31 (11 – 31)
50% hemolytic complement activity (U/mL)	> 65 (29 – 47)
Haptoglobin (mg/dL)	< 10
ADAMTS13 activity (%)	52 (> 0.78)
ADAMTS13 inhibitor (BU/mL)	0.5 (< 0.5)
Direct Coombs	–
Urinalysis	
Occult blood	3+
Protein (g/gCr)	6.23
N-acetyl-D-glucosaminidase (U/gCr)	21.0 (0.9 – 2.4)
β-2-microglobulin (μg/gCr)	9788.4 (4 – 180)
Sediments	
Red blood cells (/high-power field)	10 – 19

ADAMS13 = a disintegrin-like and metalloprotease with thrombospondin type 1 motif 13.
